# Copper Sources for Sod1 Activation

**DOI:** 10.3390/antiox9060500

**Published:** 2020-06-07

**Authors:** Stefanie D. Boyd, Morgan S. Ullrich, Amelie Skopp, Duane D. Winkler

**Affiliations:** Department of Biological Sciences, the University of Texas at Dallas, 800 W. Campbell Rd., Richardson, TX 75080, USA; sdb074000@utdallas.edu (S.D.B.); Morgan.Ullrich@utdallas.edu (M.S.U.); Amelie.Skopp@utdallas.edu (A.S.)

**Keywords:** Sod1, copper, metallo-chaperone, enzyme, metallo-enzyme, metallothionein, Ctr1, Atox1, glutathione

## Abstract

Copper ions (i.e., copper) are a critical part of several cellular processes, but tight regulation of copper levels and trafficking are required to keep the cell protected from this highly reactive transition metal. Cu, Zn superoxide dismutase (Sod1) protects the cell from the accumulation of radical oxygen species by way of the redox cycling activity of copper in its catalytic center. Multiple posttranslational modification events, including copper incorporation, are reliant on the copper chaperone for Sod1 (Ccs). The high-affinity copper uptake protein (Ctr1) is the main entry point of copper into eukaryotic cells and can directly supply copper to Ccs along with other known intracellular chaperones and trafficking molecules. This review explores the routes of copper delivery that are utilized to activate Sod1 and the usefulness and necessity of each.

## 1. Introduction

Numerous cellular processes cannot function without intracellular copper working in the active site of enzymes. The varied roles for enzyme-bound copper include, but are not limited to, energy production, signaling, and oxidative stress response [1,2,3]. Cu, Zn superoxide dismutase (Sod1) is an antioxidant enzyme that eliminates superoxide anions (O_2_^−^) from within the cell as a method of heading off the production of the more dangerous hydroxyl radicals (•OH) [4,5]. Copper serves as the cofactor necessary for this reaction [6,7,8]. The key to copper’s broad utility arises from its ability to cycle between two oxidative states: Cu(I) and Cu(II) [9]. This redox property of copper allows it to function as both an electron donor and recipient, however, this can also lead to the nonenzymatic production of hydroxyl radicals from the breakdown of hydrogen peroxide (H_2_O_2_) [2,9,10]. Sod1 homologues exist in all eukaryotic aerobes ranging from single-celled yeast to humans. With few exceptions, forms of Sod1 can be substituted between species without any phenotypic change to the organisms [11,12,13,14].

**Figure 1:**
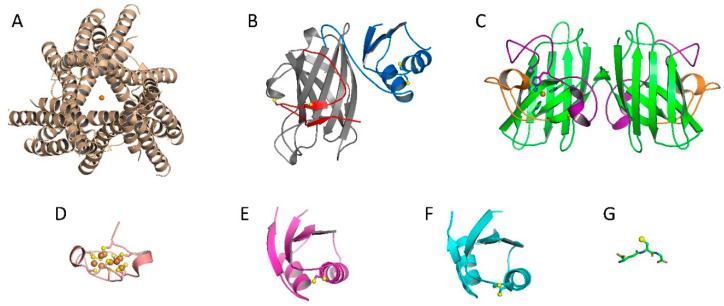
A collection of copper-binding molecules relevant to copper acquisition, regulation, and distribution to Sod1. (**A**) The copper importer Ctr1 with copper (orange sphere) bound in the channel (PDB: 6M98). (**B**) The structure of yeast Ccs, complete with D1 (blue), D2 (gray), and D3 (red). Copper binding cysteines are shown as yellow spheres (PDB: 5U9M). (**C**) Mature Sod1 dimer with the β-barrel shown in green and critical loop elements in purple (zinc loop) and orange (electrostatic loop). Active site bound copper is displayed as an orange sphere and the adjacent zinc shown in grey (PDB: 1PU0). (**D**) Copper bound MT3 domain with the coppers as orange spheres and the coordinating cysteines as yellow spheres (PDB: 1RJU). (**E**) The copper chaperone Atox1 (monomer) with the MTCxxC cysteines shown as yellow spheres (PDB: 5F0W). (**F**) A copper-binding domain (repeat 2) of the transport protein ATP7B, again with the conserved MTCxxC cysteines shown as yellow spheres (PDB: 2LQB). Notice the structural similarities between Ccs D1, Atox1, and ATP7B. (**G**) The structure of the glutathione tri-peptide, with the cysteine shown as a yellow sphere (PDB: 1AQW).

In order to prevent deleterious copper interactions, the cell utilizes a class of proteins, termed copper chaperones, to secure and deliver the necessary copper to cellular targets [9]. These proteins are known to locate the copper importers at the plasma membrane, acquire the copper as it is dispensed into the cytosol, and distribute it to specific enzymes or copper-binding proteins, thus sequestering the copper from other cellular components. There are many copper chaperones in the cell, including antioxidant 1 (Atox1) that provides copper to the transporters ATP7a and ATP7b, Cox17, which supplies copper to cytochrome c oxidase, and the copper chaperone for Sod1 (Ccs) which delivers copper exclusively to Sod1 [15,16,17,18]. Copper chaperones have been thoroughly studied for decades, however, the mode(s) of copper acquisition by these proteins remains somewhat ambiguous [19,20]. Reported copper sources for these chaperones are transporters that move copper into the various cellular compartments, copper sinks that store excess copper in the cell and other copper chaperones [21,22,23,24,25] (Figure 1).

The majority of copper enters the cytosol through the high-affinity copper uptake protein (Ctr1) [26,27]. This transmembrane import protein acquires extracellular Cu(II) from ceruloplasmin [28,29], which accounts for about 90% of copper in the blood [28], and albumin [30,31] and imports it as Cu(I). Copper reduction is likely to be facilitated by metalloreductases at the cell surface [32,33]. Studies have shown that Ccs can secure Cu(I) held by the cytosolic C-terminal domain of Ctr1 and deliver it to Sod1 [34,35]. Additionally, evidence exists that both Atox1 and Cox17 can acquire copper by way of Ctr1 [36,37] (Figure 1).

Other important cytosolic copper-binding molecules include the metallothionein superfamily of proteins (MT) and the reduced form of glutathione (GSH). MT proteins are ubiquitously expressed, highly conserved, and important for both copper and zinc homeostasis [38,39]. MT genes are upregulated, similar to those coding for Sod1, during times of oxidative stress [40]. GSH is an abundant antioxidant tripeptide present within the cytosol that, amongst other functions, can bind Cu(I) and has even been shown to deliver this cargo to Sod1 [22,41] (Figure 1).

This review will focus on the known and proposed copper transport pathways for Sod1. The movement of Cu(I) from Ctr1 to Ccs to Sod1 seems simple, yet cooperation among and transfer between copper coordinating molecules creates potential branch points and alternative routes for this pathway that are not yet fully explored [4,6,15,42,43]. Our goal is to provide new insight into the totality of copper transport pathways benefitting Sod1, and to highlight opportunities for new and exciting research in this arena.

## 2. Discussion

### 2.1. Cu, Zn Superoxide Dismutase (Sod1) and Copper Chaperone for Sod1 (Ccs1)

In 1969, McCord and Fridovich discovered an enzyme, termed erythrocuprein, which has since been renamed Sod1 [5]. It is a ubiquitously expressed antioxidant enzyme that protects the cell from the buildup of radical oxygen species through the disproportionation of superoxide to oxygen and hydrogen peroxide (2O_2_^−^ + 2H ^+^ → O_2_ + H_2_O_2_) [5]. The fully mature homodimeric form of Sod1 (Figure 1) is highly efficient, catalyzing the above reaction at or near the diffusion limit [44]. An inherent “electrostatic guidance system” composed of positively charged sites within the electrostatic loop region of Sod1 contributes to the enzyme’s catalytic efficiency (Figure 1) [4]. An arginine residue at position 143 forms the backside of the active site and is critical for both substrate attraction and nonsubstrate exclusion. Mutations at this site eliminate Sod1 activity [45]. Zinc ion (i.e., zinc or Zn(II)) binding at an adjacent site and an intra-subunit disulfide bond are also critical for both protein stability and enzymatic function (as more fully described below) [4,41].

Sod1 is abundant in the cell, found mostly in the cytoplasm, and is predominantly found in an immature state where it lacks the necessary posttranslational modifications (PTMs) required for antioxidant activity [46]. Sod1 requires three PTMs during the maturation process: (1) zinc binding at a site connected to the active site, (2) direct insertion of the catalytic copper to the active site, and (3) disulfide bond oxidation that promotes a stable homodimeric conformation [4,47,48,49,50]. Zinc is bound at a conserved loop region (i.e., zinc loop) by three histidines and an aspartic acid (H63, H71, H80, and D83). Sod1-bound copper will switch between two oxidation states during the disproportionation of superoxide (i.e., Cu(I) and Cu(II)) [5]. In the reduced form, copper is coordinated by three histidines (H46, H48, and H120) in a trigonal planar conformation [51,52]. The oxidized form of copper is coordinated by an additional histidine (H63) in a distorted square planar conformation (Figure 1) [53]. As noted above, H63 also facilitates zinc binding and is known as the “primary bridge” that links the copper and zinc sites of Sod1 [4]. A critical intra-subunit disulfide bond between C57 and C146 within each Sod1 monomer secures the loops regions making up the metal-binding sites of Sod1, which effectively excludes unwanted reactants from the active site [54,55]. Stable disulfide bonds are rare in the reducing environment of the cytosol and, when found, usually play a functional role [4].

The copper chaperone for Sod1 (Ccs) was first identified by the Culotta lab in 1997 as a protein essential for Sod1 activation in yeast [15]. The far majority of Ccs molecules across species consist of three structurally distinct domains (D1, D2, and D3) [56]. Domain 1 contains a MxCxxC copper binding motif and its fold resembles that of the copper chaperone Atox1 [48]. Proposed roles for D1 include copper acquisition from the copper importer Ctr1, copper scavenging under copper-limiting conditions and direct copper delivery to Sod1 [43]. The roles of this domain are still under debate and are likely varied between species. Ccs D2 shares both sequence and structural homology with Sod1, likely indicating that this is the main Sod1 interaction platform and a number of Sod1•Ccs crystal structures have confirmed this notion [57]. All forms of Ccs D2 lack the copper-binding site, but mammalian Ccs molecules have retained the zinc-binding site, disulfide bond, and loop elements [58]. Domain 3 is a relatively short, unstructured, yet highly conserved domain that contains a CxC copper binding motif [59]. The available evidence seems to point towards a role in copper delivery and/or formation of the disulfide bond in Sod1 [6,60]. In recent publications, we and others have demonstrated that Ccs domain 3 can form a stable β-hairpin [41,43]. Our structure shows the conserved CxC motif located near the disulfide residues and active site of Sod1, inferring a molecular mechanism for copper delivery and disulfide bond formation [41]. More recent work has revealed that Ccs prefers a completely immature form of Sod1 (E,E-Sod1SH) and each step of maturation decreases the affinity of the Sod1•Ccs interaction [61]. In addition to delivering copper for Sod1, Ccs binding increases the affinity and zinc occupancy of Sod1, of which D3 is required [60].

### 2.2. Ccs-Mediated Sod1 Maturation

The traditional understanding of Ccs-mediated Sod1 maturation highlights the well-established roles for copper delivery and disulfide bond formation in Sod1 [47]. We and others have expanded upon this mechanism to include roles for Ccs that include: Sod1 folding, facilitated site-specific zinc acquisition and the formation of a copper drop-off point or “entry-site” upon Sod1 binding [41,60,62]. These additional properties separate Ccs from all other known copper chaperones, and thus, Ccs is better characterized as a multifunctional chaperoning molecule with roles in each level of Sod1 maturation.

Our “entry-site” model for Ccs-mediated copper delivery proposes that Cu(I) is delivered from the CxC copper-binding motif in Ccs D3 to site formed near the Sod1•Ccs heterodimeric interface [41]. Observation of the Sod1-Ccs crystal structure published previously by our lab reveals a strong interaction between Sod1 and a B-hairpin formed by Ccs D3 [4]. Further spectroscopic analysis of the purified protein complex showed that a copper atom was coordinated at this entry site [36]. This interaction was also biochemically analyzed with in vitro functional and mutational assays, which suggest that the site is formed via residues in both Sod1 (H120 and C57) and Ccs (C231, yeast sequence). Intercalation of the Ccs D3 β-hairpin under the Sod1 disulfide loop exposes an electropositive path that likely attracts a superoxide or hydroxide molecule to the coordinated copper and accelerates sulfenylation of the adjacent C146 on Sod1. The existence of the sulfenyl intermediate was confirmed by Western blot with an antibody specific to sulfenyl modified cysteines [36]. Key disulfide exchange reactions promoted by the sulfenic acid group on C146 ends with the formation of the critical C57-C146 Sod1 disulfide bond and releases of the copper into the active site. Disulfide bond formation in Sod1 terminates interaction with Ccs and promotes its own homodimerization, where the mature enzyme can now perform its normal antioxidant functions [4,41].

An alternative model has been proposed by the laboratory of Lucia Banci. Here, Ccs D1 is primarily responsible for copper acquisition and delivery to the Sod1 active site [6], though, the structural details of this proposed conformational switch are currently undefined. The singular role for Ccs D3 is the transfer of a preformed disulfide bond to Sod1. As opposed to the “entry-site” model, this version is a distinct two-step process where copper delivery and disulfide bond formation are separate, though closely related, functions of Ccs. This model is based on experimental evidence obtained using in-cell NMR to measure metal transfer.

Until very recently, the role, if any, Ccs plays in Sod1 zinc acquisition had been essentially ignored. New evidence, from our lab and others, has illustrated multifunctional or molecular chaperoning roles for Ccs [60,63]. We have highlighted that Ccs functions in both copper delivery and facilitating high-affinity Zn(II)-binding of Sod1 [60]. Data show that Ccs binding to Sod1 increases both affinity and occupancy of zinc at the zinc-binding site in Sod1. This is likely due to Ccs stabilizing a beneficial conformation of the zinc loop; an extension of the disulfide loop that makes up a section of the interaction interface between Sod1 and Ccs (Figure 1). Interestingly, it has also been shown that Ccs binding alone (i.e., without copper delivery) can resolve mis-metalation events where zinc is initially found in the active site. However, some pathogenic Sod1 mutants that cause an inherited form of amyotrophic lateral sclerosis (fALS) seemingly counteract these processes [62].

### 2.3. Ccs-Independent Sod1 Maturation

While highly conserved across all eukaryotic organisms, there are some divergencies among the group of Sod1 molecules [64]. One important distinction is the existence of an additional pathway for Sod1 maturation; the Ccs-independent pathway is necessitated by a simple amino acid change (e.g., proline substitution) between mammals and other lower eukaryotes [65,66]. Indeed, human Sod1 expressed in ΔCcs/ΔCcs mice retains ~25% activity, thus indicating a pathway for Sod1 maturation liberated from Ccs [7]. Another interesting anomaly is *Caenorhabditis elegans*, where no Ccs exists, yet the population of Sod1 molecules are found to have their complete assortment of posttranslational modifications and are fully active [67]. For most other eukaryotes, including yeast, Sod1 maturation requires Ccs [19].

Potential copper sources for the Ccs-independent Sod1 maturation pathway have been proposed that include, but are not limited to, the reduced form of glutathione (GSH), metallothioneins (MTs) and other known copper chaperones [65,68,69]. Nevertheless, there lacks solid evidence to indicate a clear mechanism for this pathway. GSH seems one of the most likely sources as it has been shown to acquire copper from the Ctr1 importer and is present in large quantities within the cytosol [65]. The mechanism of a potential GSH-mediated Sod1 maturation event is unclear, to say the least.

Leitch et al. have found that the kinetics of Sod1 activation and the copper source is identical despite the differing roles for Ccs (e.g., completely dependent, independent, or partially dependent on Ccs) [66]. In contrast, the mechanism of disulfide bond formation for Sod1 is strikingly different and contingent upon the role that Ccs plays in its activation. Ccs dependent disulfide bond formation relies on available oxygen species, yet, inexplicably, the Ccs-independent pathway does not [42,65,66]. Ensuing sections will delve into the current literature and preliminary models for these pathways.

### 2.4. Copper Acquisition by Ccs via Ctr1

The mechanism of Ccs-mediated Sod1 maturation has been studied extensively, as evidenced by the dense literature and multiple models for Ccs action [4]. On the other hand, the source(s) of copper for this process and connections to other copper delivery pathways have not been fully explored. The major port of entry for all copper trafficking is the copper importer Ctr1 [34]. From this point, it is proposed that the intracellular trafficking molecules acquire and then depart towards target delivery. Is there a pecking order among the trafficking molecules? Are their intermediaries in the acquisition or delivery processes? How about during times of stress or copper variability? We hope to address these unresolved questions while centering our discussion around the routes of copper delivery to Sod1.

The Ctr-family is a functionally, but not structurally, conserved group of transmembrane copper transporters present across eukaryotic organisms, of which Ctr1 is the most prevalent [70,71,72,73,74,75,76]. Ctr proteins import copper from the extracellular environment into the cytosol. For humans and other select species, more than one copper transporter has been identified [25,77,78,79]. Ctr2 functions alongside Ctr1 as a low-affinity copper transporter, a regulator of micropinocytosis, and modulator of Ctr1 positioning within the plasma membrane. Ctr2 influences Ctr1 membrane location by promoting a truncated form of Ctr1 that is a target of the endo-lysosomal pathway [24,33,80].

The human form of Ctr1 is comprised of a large extracellular domain, three transmembrane helices, and a short C-terminal cytosolic tail that harbors the intracellular copper-binding HCH motif [26,81,82,83]. The functioning transporter is a trimeric conical channel; a 9 Å opening faces the extracellular milieu that widens towards the cytosol (22 Å) [32,84,85]. The N-terminal ecto-domain is glycosylated (both N- and O-linked) and these modifications likely impact Ctr1 stability and plasma membrane localization [27,86,87]. While the exact mechanism of copper transfer is not fully understood, ATP-driven transport of the metal has been excluded due to the lack of an ATP-hydrolyzing domain [88]. Histidine residues lining the inside of the transmembrane domain regulate Cu(I) import, which is roughly 80% of all copper brought into the cell [32,81]. While fungal copper transporters work in conjunction with copper reductases, little evidence exists for a similar setup in mammalian systems [89,90]. Further research is needed to elucidate the mechanism of copper reduction prior to Ctr1-mediated copper import.

A C-terminal HCH motif on Ctr1 acts as a sink for Cu(I) ions and is suggested to be the site of hand-off for copper chaperones and other copper binding molecules [21,91]. Docking studies between Atox1 and the C-terminal domain of Ctr1 (Ctr1c) show that Cu(I) can be donated directly from Ctr1 to Atox1 [68]. Related work by the Unger group suggests that Ccs1 and Atox1 can associate with the plasma membrane, thus providing a means for targeted interaction between transporter and chaperone [21,92]. Relatedly, Kaplan and colleagues demonstrated that Ctr1 could hand copper to the reduced form of glutathione (GSH), which is present in high concentrations within the cytosol and can act as a copper reservoir [92].

Our lab has recently shown that the Cu(I)-bound form of Ctr1c can form a stable complex with Ccs [34]. Only the addition of immature Sod1 (i.e., copper-free and disulfide reduced) to this complex can separate the copper-mediated Ctr1c•Ccs complex. The reaction produces a mature Sod1 molecule (i.e., copper bound and disulfide oxidized) that is fully active. When a C146A mutation is introduced to Sod1 that prevents both copper delivery and disulfide bond formation, a stable Ctr1c•Ccs•Sod1 trimeric complex is observed. These results help to confirm that copper mediates a stable interaction between Ctr1 and Ccs and that localized activation of Sod1 terminates all interactions [34]. Interestingly, Ctr1 is not obligatory for Sod1 to access copper [93]. Thus, what are other potential routes of copper delivery for Ccs-mediated and Ccs-independent Sod1 maturation?

### 2.5. Involvement of other Chaperones and Metalloproteins

#### 2.5.1. Atox1

Atox1 delivers copper to the secretory pathway and was one of the first members of the copper chaperone family [94]. Atox1 comprises a small single-domain with a ferredoxin-like fold and a conserved MTCxxC copper binding motif, which it shares with its targets ATP7A and ATP7B [2,95,96]. ATP7A/B are P-type ATPase copper transporters that remove excess copper from the cytosol, thus protecting the cell from elevated copper levels [1]. Atox1 also provides the copper essential for metalation of secreted proteins such as ceruloplasmin, a copper-carrying ferroxidase in the blood [97].

As stated previously, both Ccs and Atox1 are capable of interacting with lipid bilayers and this may facilitate interaction with Ctr1 [35,98]. Localization of the plasma membrane may also enable copper exchange between the two chaperones. Ccs D1 and Atox1 share both structural and sequence homology, which allots similar copper-binding affinities [99]. Despite their similarities, Atox1 cannot fulfill the role of Ccs in the cell [100]. Wittung-Stafhede and colleagues have shown that Ccs can retrieve Cu(I) from Atox1 by coupled size-exclusion chromatography and copper transfer assays [68]. While Atox1 can access copper though Ctr1, its additional role in removing excess copper from the cytosol and its ability to transfer copper to Ccs provides another likely route for copper in Ccs-mediated Sod1 maturation [16]. These findings show the cross-reactivity and interconnectedness of the cellular copper transfer machinery [68].

#### 2.5.2. MTs

The metallothionein family encompasses a group of small cysteine-rich proteins involved in metal homeostasis and detoxification [39]. Most MTs buffer zinc, however, their thiolate clusters can act as a copper sink if cellular copper concentrations are high [23,101]. Though all isoforms bind copper, when MT1 and MT2 are isolated from cells, each is bound solely to zinc [39,102]. MT3 mostly occupies the central nervous system and is isolated with both zinc and copper bound, likely because of its complex biological roles in these cells [40]. There lacks extensive research concerning copper transfer between MTs and Sod1 or Ccs. However, MTs have been shown to influence zinc binding by Sod1 [103]. MTs bind copper with a high affinity (average 10^−19^ M), thus a potential mechanism for hand-off to copper chaperones, carriers, or enzymes is not obvious, but could involve oxidation of the coordinating cysteines by molecules such as superoxide [40]. Crosstalk between these classes of molecules is quite likely as ever-changing cellular conditions require the activity of specific target proteins, including Sod1 [69,104].

#### 2.5.3. Glutathione

One major copper recipient from Ctr1 is the reduced form of glutathione (GSH), which is present within the cell at low millimolar concentrations in order to maintain the redox status of the cytosol while also functioning as an intracellular copper sink. A number of independent groups have shown that GSH can supply copper to chaperones such as Ccs [22,92,105]. GSH likely serves as a general copper reservoir for cytosolic copper-binding proteins. The relatively low copper affinity emphasizes the role of GSH as an upstream hub for copper delivery to multiple downstream targets. In accordance, when monitoring copper uptake via Ctr1, only GSH depletion impaired cellular copper uptake, while knock out of copper chaperones such as Atox1 or Ccs did not have a pronounced effect [92].

In mammals, it has been demonstrated that Sod1 can be activated in a Ccs-independent manner and that GSH plays an essential, if not well defined, role in this pathway [22]. For lower eukaryotes (e.g., yeast), GSH cannot unilaterally activate Sod1, but GSH has shown the ability to provide copper for activation in the presence of Ccs [34,41]. Here, Cu(I)-GSH was added to a pre-made Sod1•Ccs complex, where the complex was void of copper and resulted in copper transfer and complete maturation of Sod1. It was noteworthy that the copper acquisition role of Ccs D1 was essentially bypassed as the process works with a mutant version of Ccs where the MxCxxC copper binding motif cysteines were changed to alanines. Additionally, the removal of Ccs D3 prohibited the Sod1 maturation process. We have proposed that Ccs D3 forms a copper “entry-site” or drop off point along with the disulfide cysteines of Sod1 near the interaction interface. Without this site, copper delivery to Sod1 and disulfide bond formation cannot occur [41].

## 3. Conclusions and Perspectives

**Figure 2:**
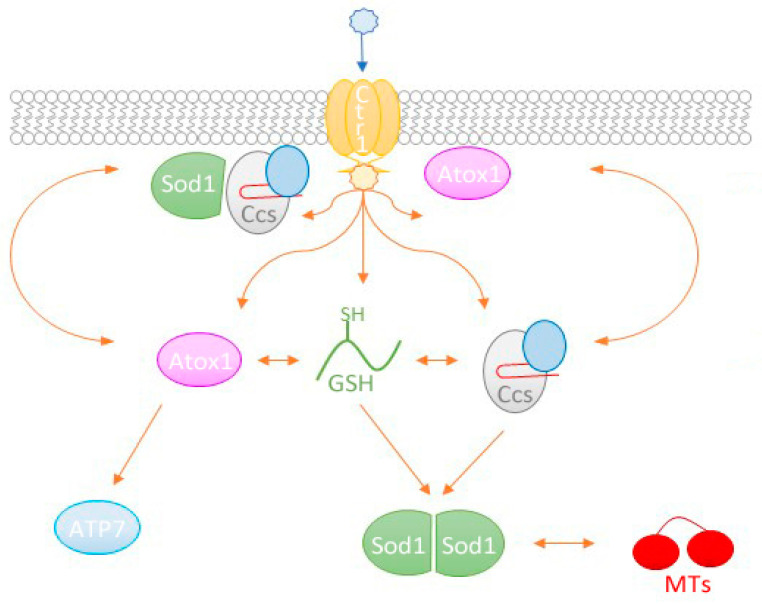
Potential routes of delivery between the copper importer Ctr1 and Sod1. Extracellular copper is commonly carried as Cu(II) (blue star) and must be reduced to Cu(I) (yellow star) for import by Ctr1. From here, the copper has numerous paths that can be taken based upon cellular conditions and availability of carrier molecules (orange arrows). Both Ccs and Atox1 have shown the propensity to associate with lipid bilayers, which may facilitate copper acquisition from Ctr1. Additionally, Atox1 and Ccs have been demonstrated to directly exchange copper with each other before delivery to their targets (ATP7A/B and Sod1, respectively). Past work in the field has also shown that the reduced form of glutathione (GSH) can act as a copper acceptor from Ctr1, exchange that copper with other copper chaperones while also delivering its copper cargo directly to immature Sod1. Further evidence has suggested that metallothioneins (MTs) may be able to exchange copper(s) with other copper-binding proteins, like Sod1, likely under conditions of oxidative stress. The colors of molecules and domains in this figure attempt to mimic those in Figure 1.

Copper is tightly regulated due to the vital roles it plays within the cell, but also because of its potential for adverse redox activity [2]. To maintain homeostasis, cells have evolved an intricate copper distribution center to efficiently direct copper delivery [3]. For the vast majority, copper’s journey in the mammalian cell begins when it is imported by Ctr1 [26]. This transporter moves copper across the plasma membrane and uses its cytosolic C-terminal tail (Ctr1c) to hold the copper until it can be picked up by a chaperoning protein, such as Atox1, Ccs or a sequestering molecule such as GSH or MT protein (Figure 2) [21,27]. It is very likely that these copper carriers participate in extensive crosstalk and regulation, which maintains copper homeostasis and allows for quick response to cellular changes [104].

For years, accumulating evidence has indicated that copper-dysregulation plays a significant role in the development of several neurodegenerative diseases [3]. As previously described, the import of copper by Ctr1 plays a pivotal role in the copper supplementation of intracellular and secreted molecules [32]. Both Menkes and Wilson’s Disease are caused by a disruption in copper homeostasis due to mutations in the transmembrane copper pumps ATP7A and ATP7B, respectively [3]. A diverse set of mutations in Sod1 causes an inherited form of the fatal neurodegenerative disease fALS [106,107]. A role for Ccs in Sod1-linked fALS is gaining momentum as immature forms of the Sod1 protein make up a large proportion of the aggregates found in the motor neurons of mice expressing pathogenic forms of Sod1 and from the autopsies of ALS patients [108].

Given that copper regulation is important to cellular function and that dysfunction often leads to disease, therapeutics targeting copper maintenance could be useful in treatment [3,9]. For instance, diacetylbis(N(4)-methylthiosemicarbazonato)-copper(II) (Cu(II)-ATSM) is currently used for imaging tumor hypoxia, but a recent discovery indicates that Cu(II)-ATSM could be a potential therapeutic agent in the treatment of ALS [109]. Potential future treatments will rely on our understanding of copper trafficking and homeostasis. Insights gained from the thorough understanding of metal transfer between Ccs and Sod1 provide a framework for future studies.

Intracellular copper transport has been studied extensively for many years. However, key elements of copper trafficking are still unknown. Ctr1 has been investigated for nearly 30 years [110], but its mode of copper import, initial copper acquisition and reduction to Cu(I), and possible interactions with copper carriers has not been fully elucidated. Upon transfer from Ctr1 to designated copper coordinating molecule, the complex interplay between these key players of intracellular copper delivery still harbors unresolved questions. Do chaperones directly retrieve copper from Ctr1, or do they acquire copper from a Cu(I)-GSH intermediary? Does chaperone scaffolding with the plasma membrane support copper acquisition and/or possible delivery to the target? Does the availability of copper influence the mode of copper acquisition by chaperones, carriers and their target enzymes? More investigation, including biophysical, biochemical, and cellular approaches, is required to map out this intricate intracellular copper network to improve our understanding of these elaborate copper delivery mechanisms and, ultimately human health.
